# Co-transcriptional production of programmable RNA condensates and synthetic organelles

**DOI:** 10.1038/s41565-024-01726-x

**Published:** 2024-07-30

**Authors:** Giacomo Fabrini, Nada Farag, Sabrina Pia Nuccio, Shiyi Li, Jaimie Marie Stewart, Anli A. Tang, Reece McCoy, Róisín M. Owens, Paul W. K. Rothemund, Elisa Franco, Marco Di Antonio, Lorenzo Di Michele

**Affiliations:** 1 Department of Chemistry, Imperial College London, London, UK; 2 fabriCELL, Imperial College London, London, UK; 3 Department of Chemical Engineering and Biotechnology, University of Cambridge, Cambridge, UK; 4 The Francis Crick Institute, London, UK; 5 Department of Bioengineering, University of California at Los Angeles, Los Angeles, CA USA; 6 Department of Computing and Mathematical Sciences, California Institute of Technology, Pasadena, CA USA; 7 Department of Mechanical and Aerospace Engineering, University of California at Los Angeles, Los Angeles, CA USA

**Keywords:** DNA nanotechnology, Nanobiotechnology

## Abstract

Condensation of RNA and proteins is central to cellular functions, and the ability to program it would be valuable in synthetic biology and synthetic cell science. Here we introduce a modular platform for engineering synthetic RNA condensates from tailor-made, branched RNA nanostructures that fold and assemble co-transcriptionally. Up to three orthogonal condensates can form simultaneously and selectively accumulate fluorophores through embedded fluorescent light-up aptamers. The RNA condensates can be expressed within synthetic cells to produce membrane-less organelles with a controlled number and relative size, and showing the ability to capture proteins using selective protein-binding aptamers. The affinity between otherwise orthogonal nanostructures can be modulated by introducing dedicated linker constructs, enabling the production of bi-phasic RNA condensates with a prescribed degree of interphase mixing and diverse morphologies. The in situ expression of programmable RNA condensates could underpin the spatial organization of functionalities in both biological and synthetic cells.

## Abstract (web summary)

Controlling RNA and protein condensation is helpful in synthetic biology. Here the authors show programmable assembly of synthetic RNA nanostructures into designer membrane-less organelles that selectively recruit ligands via protein-binding aptamers.

## Main

Membrane-less compartmentalization sustained by biomolecular condensates is recognized as a primary regulatory mechanism in cells^1–4^. By co-localizing nucleic acids, enzymes and metabolites, membrane-less organelles (MLOs) such as nucleoli, Cajal bodies and stress granules are believed to regulate biogenesis, transcription, post-transcriptional modification and degradation^4–7^, while pathological condensates have been linked to neurodegeneration^8,9^.

The ability to express ‘designer condensates’ with prescribed properties would be valuable to program cellular behaviour^10,11^ and engineer synthetic cells^12^. Remarkable examples based on peptides^10,11^ or natural RNA repeat sequences^9,13,14^ and riboswitches^15,16^ have highlighted the feasibility of this concept. The generality of these strategies, however, is hampered by the challenges of protein engineering and the limited programmability of natural RNA constructs.

Leveraging nucleic acid nanotechnology^17–19^, in this paper we introduce a systematic method for expressing designer biomolecular condensates from synthetic RNA nanostructures. Our elementary motifs consist of star-shaped junctions, or nanostars, which fold co-transcriptionally and assemble driven by selective base-pairing interactions, forming up to three co-existing but fully distinct condensate types. Expressing the condensates in synthetic cells generates MLOs with controlled size, number, morphology and composition. Finally, including RNA aptamers enables selective capture of small molecules and proteins, imitating the ability of natural MLOs to recruit clients.

Because the RNA nanostars are transcribed in situ from DNA templates, our platform could be directly applied to express synthetic MLOs in living cells, besides its immediate use in synthetic cells. In this context, exploring the design space of RNA nanostars will allow for fine-tuning of condensate properties^20^.

## RNA nanostar design and co-transcriptional condensation

**Fig. 1:**
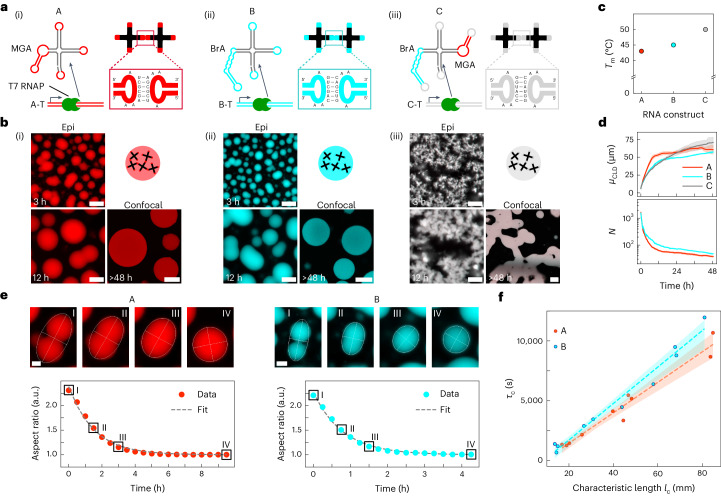
Condensation of co-transcriptionally folding RNA nanostars. **a**, Structure of the RNA motifs. An A-type RNA nanostar includes an MGA (i), B-type includes a BrA (ii) and C-type includes both aptamers (iii). Variants feature mutually orthogonal, self-complementary (palindromic) KLs, whose sequences are shown in the insets. RNA nanostars are transcribed from linear dsDNA templates by T7 RNAP. **b**, Epifluorescence and confocal micrographs showing condensate formation and coarsening for all three designs in **a** at different timepoints of an in vitro transcription reaction. Epifluorescence micrographs have been linearly re-scaled to enhance contrast (Supplementary Methods 2). Pristine micrographs are shown in Supplementary Fig. 5. Scale bars, 50 μm. Timestamps are reported with respect to the start of time-lapse imaging (Methods and Supplementary Table 5). **c**, Melting temperatures (*T*_m_) of A–C condensates, determined as discussed in Methods, Supplementary Methods 2, and Supplementary Figs. 8 and 9. **d**, Top: mean of the CLD, *μ*_CLD_. Bottom: average number of condensates per microscopy field of view, *N*, as a function of time. Full CLDs, as extracted from image segmentation, are shown in Supplementary Fig. 17 (Supplementary Methods 2). *N* is not computed for system C, which does not form discrete aggregates. Data are shown as mean (solid lines) ± s.d. (top) or s.e. (bottom) (shaded regions) of three field of views within one sample. **e**, Top: epifluorescence micrographs (contrast enhanced) depicting coalescence events for A (left) and B (right) condensates. Scale bars, 15 μm. Bottom: time-dependent aspect ratio of the condensates above, computed as the ratio between major and minor axes of the best-fit ellipse. The dashed line shows an exponential fit with decay constant *τ*_c_. **f**, *τ*_c_ against the characteristic size (*l*_c_) of A and B condensates undergoing coalescence. Linear regression yields inverse capillary velocities *μ*/*γ* = 127.4 s μm^−1^ and 152.3 s μm^−1^ for A and B condensates, respectively^46^ (Supplementary Methods 2). The dashed lines indicate best fits, with 95% confidence intervals shown as shaded regions. Transcription and coalescence events occurred at constant 30 °C temperature (Methods). Source data

The four-armed nanostars consist of a single RNA strand that folds co-transcriptionally into the intended star-like shape, inspired by well-characterized DNA nanostars^21–23^ (Fig. 1a). The RNA motifs interact via self-complementary HIV-type kissing loops (KLs) present at the end of each arm^24^, rather than via the single-stranded (ss) overhangs or hydrophobic modifications adopted for DNA designs^21–23^. Similar KLs have been shown to facilitate condensation in bacterial riboswitches^15,16^. We tested three RNA-nanostar designs, labelled A, B and C, featuring mutually orthogonal KL sequences (Fig. 1a). In designs A and B, one of the double-stranded RNA (dsRNA) arms includes a fluorescent light-up aptamer (FLAP): malachite green aptamer (MGA) for A^25,26^ and Broccoli aptamer (BrA) for B^27^ (Fig. 1a(i),(ii)). FLAPs yield a fluorescent signal upon binding their cognate fluorophores (malachite green (MG) for MGA and 3,5-difluoro-4-hydroxybenzylidene imidazolinone (DFHBI) for BrA), enabling characterization via fluorescence microscopy and fluorimetry (Supplementary Fig. 1). Design C includes both MGA and BrA in non-adjacent arms (Fig. 1a(iii)). The arms not hosting FLAPs are 25 base-pairs long, and separated by an unbound uracil for flexibility^28^. The motifs were transcribed with T7 RNA polymerase (T7 RNAP) from double-stranded DNA (dsDNA) templates, labelled as A-T, B-T and C-T for designs A, B and C, respectively (Supplementary Fig. 2). Denaturing polyacrylamide gel electrophoresis confirms the expected electrophoretic mobility for most transcripts, with small amounts of truncated and over-elongated products^29,30^ (Supplementary Fig. 3). Native agarose gel electrophoresis suggests that transcripts retain the intended folded monomeric conformation, rather than producing misfolded multimers^15,16^ (Supplementary Fig. 4). See Methods for sequence design and transcription protocols, and sequences in Supplementary Tables 1–4.

The micrographs in Fig. 1b show that all three designs formed aggregates when transcribed in vitro (see also Supplementary Figs. 5 and 6 (top), and Supplementary Video 1 (top)). Variants A and B formed condensates that nucleated and grew, with frequent coalescence events, indicating a liquid state. The condensates were roughly spherical and did not substantially wet the glass substrate (Supplementary Fig. 7), consistent with the evidence of Brownian motion (Supplementary Video 1). Conversely, design C formed a gel-like percolating structure that failed to produce discrete condensates but still grew over time. The higher apparent viscosity of design C compared with A and B correlates with the melting temperature of the materials, which is highest in C, followed by B and A (Fig. 1c, Supplementary Figs. 8 and 9, and Supplementary Video 2). All aggregates showed the intended fluorescence output, namely, in the MGA channel for A (red), the BrA channel for B (cyan) and both channels for C (white).

Degradation and bubble formation was observed in the condensates over time, ascribed to environmental nucleases^31^ or photo-degradation (Supplementary Fig. 5). Bubbling was most prominent for A, consistent with the lower melting temperature and consequent expectation that less damage would be required to trigger disassembly. Sequence differences between the constructs may also influence their susceptibilities to degradation.

The specificity of KL interactions was confirmed by non-sticky, control designs—$\bar{{{{\rm{A}}}}}$ and $\bar{{{{\rm{B}}}}}$—where KLs were replaced with scrambled sequences. These did not yield condensates and produced only diffused fluorescence (Supplementary Figs. 6 (bottom) and 10, and Supplementary Video 1 (bottom)).

The evidence that condensation requires KL complementarity suggests that non-specific, cation-dependent, phase-separation mechanisms^32^ are not dominant. To further elucidate the role of cations, we characterized the stability of A and B condensates upon buffer replacement (Supplementary Fig. 11). Condensates remained stable after 24 hours in Tris-EDTA buffer supplemented with 5 mM or 10 mM MgCl_2_, but disassembled in phosphate-buffered saline, consistent with previous evidence that divalent cations stabilize KL interactions^33^.

We assessed the effect of crowding agents, often introduced to aid RNA condensation in vitro^34,35^, on co-transcriptional assembly of RNA nanostars (Supplementary Fig. 12). When including 25% v/v polyethylene glycol (PEG) 200, we observed a reduction in condensate size in both A and B systems, consistent with previous observations that PEG 200 reduces the T7 RNAP transcription rate^36^. Non-binding variants $\bar{{{{\rm{A}}}}}$ and $\bar{{{{\rm{B}}}}}$ remained soluble in the presence of PEG, which is thus insufficient to trigger non-specific condensation.

Bulk fluorimetry was used to monitor the rate of synthesis of the RNA constructs (Supplementary Fig. 13). All designs initially showed a rapid signal increase, whose rate scaled (nearly) linearly with template concentration (Supplementary Figs. 14 and 15). This initial phase was followed by a plateau or slower growth, probably due to loss of polymerase activity and/or nucleotide depletion^37–39^. A peak, ascribed to aggregate sedimentation, was noted for sticky motifs (A, B and C) but not for non-sticky designs ($\bar{{{{\rm{A}}}}}$ and $\bar{{{{\rm{B}}}}}$). Differences in plateauing behaviours could derive from variations in the kinetics of aptamer folding and/or complexation with fluorophores^40–42^. Epifluorescence micrographs reveal that, after an initial transient, the ratio between nanostar concentration in the bulk and within the condensates reached a plateau, indicative of a steady state between dilute and condensed RNA phases (Supplementary Fig. 16 and Supplementary Methods 2).

We gained further insights on condensate growth and coarsening dynamics from chord-length distribution (CLD) analysis of epifluorescence micrographs^43–45^ (Supplementary Fig. 17 and Supplementary Methods 2). The CLD provides a time-dependent picture of condensate length scales in a way that is agnostic of their shape, and is thus equally meaningful for the branched C-type structures and the compact A and B condensates.

Figure 1d (top) shows the time evolution of the mean of the CLD, *μ*_CLD_, which is useful as a proxy for the typical condensate size. For all designs, *μ*_CLD_ rapidly increased at early stages, probably sustained by the active transcription leading to monomer addition (Supplementary Figs. 13 and 16). For A and B, frequent coalescence events also contributed to the increase in *μ*_CLD_, reflected by a steep decrease in the number of condensates (*N*; Fig. 1d (bottom)). Coalescence appears to occur more readily in A, given the steeper decrease in *N* and increase in *μ*_CLD_ compared with B. Supplementary Video 1 suggests that early coalescence may be driven by neighbouring condensates touching as they grow through monomer addition, aided by Brownian motion. Consistently, both the size and number of A and B condensates plateaued when transcription slowed (Supplementary Fig. 13). In C aggregates, the increase of *μ*_CLD_ continued at later times, driven by the slow coarsening of the percolating RNA network (Fig. 1b(iii) and Supplementary Video 1).

The coalescence dynamics of A and B condensates can be further analysed to determine the inverse capillary velocity of the RNA phases, namely, the ratio between their viscosity (*μ*) and surface tension (*γ*)^46–48^ (Supplementary Methods 2). As summarized in Fig. 1e,f, we find *μ*/*γ* = 127.4 s μm^−1^ and *μ*/*γ* = 152.4 s μm^−1^ for A and B condensates, respectively. These values are significantly higher compared with DNA-nanostar condensates (0.9 s μm^−1^ to 26.3 s μm^−1^; refs. ^46–48^), but compatible with the broad range reported for protein-based and biological condensates (~10^−2^ s μm^−1^ to ~10^2^ s μm^−1^; refs. ^49,50^). Fluorescence recovery after photobleaching (FRAP; Supplementary Methods 4), performed using dyes covalently linked to the RNA (Methods), revealed lack of recovery over >500 s for both A and B (Supplementary Fig. 18), suggesting a higher viscosity of RNA-nanostar condensates compared with their DNA counterparts^46–48^. When performing FRAP using the embedded FLAPs, both A and B condensates showed rapid fluorescence recovery, probably due to exchange of dyes with the bulk^42^ (Supplementary Fig. 18).

## Orthogonal RNA condensates

**Fig. 2:**
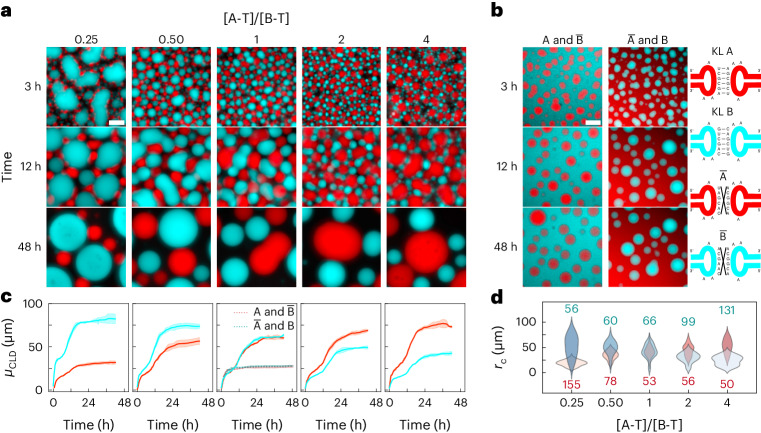
Co-transcribed orthogonal RNA nanostars form immiscible condensates of controlled size. **a**, Epifluorescence micrographs of binary systems of A and B RNA nanostars (see Fig. 1) at various timepoints during the transcription transient. Different ratios between the concentrations of the two templates, A-T and B-T, are tested, while keeping [A-T] + [B-T] constant. **b**, Epifluorescence snapshots analogous to **a**, but where either A or B is replaced by its non-sticky variant, namely, $\bar{{{{\rm{A}}}}}$ or $\bar{{{{\rm{B}}}}}$. Sketches on the right-hand side show examples of scrambled non-binding KL sequences. **c**, Time evolution of *μ*_CLD_ (Supplementary Methods 2), for samples in **a** and **b**, the latter shown in the central panel as dashed lines. See Supplementary Fig. 23 for full CLDs. Red and cyan curves are relative to A and B condensates, respectively. Data are shown as mean (solid/dashed lines) ± s.d. (shaded regions) of three fields of view within one sample. **d**, Distribution of the radii, *r*_c_, of A (red) and B (cyan) condensates as a function of the template concentration ratio [A-T]/[B-T]. Epifluorescence micrographs in **a** and **b** have been linearly re-scaled to enhance contrast (Supplementary Methods 2). Pristine micrographs are shown in Supplementary Figs. 19 and 21. All scale bars, 50 μm. Timestamps are reported with respect to the start of time-lapse imaging (Methods and Supplementary Table 5). Source data

KL orthogonality enables the simultaneous transcription of A and B designs, which readily formed distinct, co-existing condensates (Fig. 2a, Supplementary Figs. 19 and 20, and Supplementary Video 3), confirming the negligible influence of base-pairing-independent condensation pathways^32^. Consistently, if one of the RNA motifs was rendered non-sticky, condensates of one species co-existed with dispersed RNA nanostars of the other (Fig. 2b, Supplementary Figs. 21 and 22, and Supplementary Video 4).

The relative size of A and B condensates can be controlled by tuning the ratio between the concentrations of the corresponding DNA templates ([A-T] and [B-T]). Relative-size control is demonstrated visually (Fig. 2a, and Supplementary Figs. 19 and 20), through time-dependent *μ*_CLD_ analysis (Fig. 2c and Supplementary Fig. 23), and through the distribution of the final condensate radii, *r*_c_, (Fig. 2d and Supplementary Methods 2). Condensate numbers anticorrelate with their size. For instance, many small A-type condensates were formed when [B-T] > [A-T] (Supplementary Fig. 24).

Condensate growth occurred in two stages in all sticky A and B systems: after an initial increase, a brief intermediate plateau was reached, followed by another growth phase before saturation (Fig. 2c). This behaviour was not observed in single-component systems (Fig. 1d), nor in binary systems with one sticky and one non-sticky species (Fig. 2c, middle). Supplementary Videos 3 and 4 reveal that the intermediate plateau was reached when same-type condensates became temporarily unable to coalesce due to being ‘caged’ by neighbouring condensates of the opposite type. Coalescence events that still managed to occur, however, reduced lateral crowding given that merged condensates occupy less space in the horizontal plane, unjamming the system and accelerating coalescence. In fact, condensates reached larger dimensions in A and B systems (Fig. 2c) compared with $\bar{{{{\rm{A}}}}}$ and B, and A and $\bar{{{{\rm{B}}}}}$ mixtures (Fig. 2b and Supplementary Fig. 23), indicating that steric encumbrance from non-binding condensates of the opposite phase ultimately facilitates coalescence. Two-step decreasing trends, consistent with those seen in *μ*_CLD_, were observed in condensate numbers (Supplementary Fig. 24).

Addition of 25% v/v PEG 200 induced non-specific affinity between A and B condensates^51,52^ (Supplementary Fig. 12), leading to extended networks of small alternating A and B domains reminiscent of colloidal gelation^53^.

Condensate co-assembly is also possible with three RNA species A, B and C (Supplementary Figs. 25 and 26, and Supplementary Video 5. After allowing sufficient time for relaxation, all species formed spherical condensates, including C, which was unable to do so in single-component samples (Fig. 1b(iii)). The difference in morphology is probably due to A and B stars hindering the formation of a percolating C network in favour of smaller aggregates that relax more readily.

## RNA MLOs in synthetic cells

**Fig. 3:**
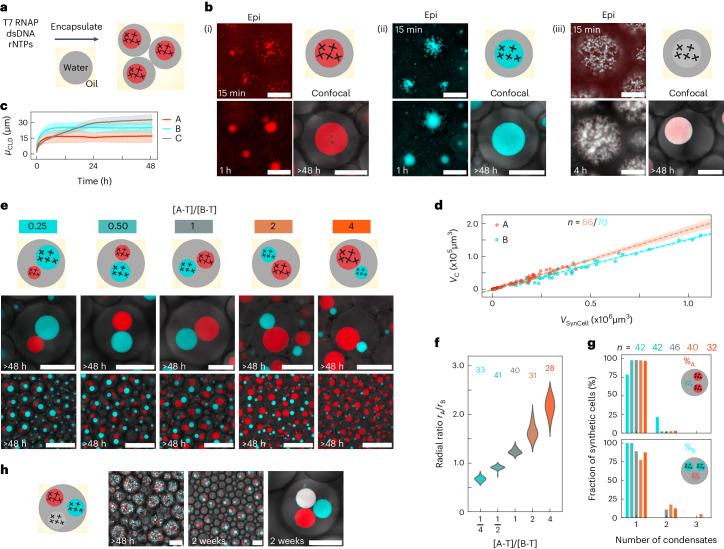
Membrane-less RNA organelles expressed in synthetic cells. **a**, Diagram showing MLOs formed in synthetic cells consisting of W/O emulsion droplets encapsulating transcription machinery, ribonucleotide triphosphates (rNTPs) and DNA templates. **b**, Epifluorescence and confocal micrographs showing MLO formation over time in synthetic cells expressing A-type (i), B-type (ii) and C-type (iii) RNA nanostars (see Fig. 1). Epifluorescence micrographs have been linearly re-scaled to enhance contrast (Supplementary Methods 2). Pristine images are shown in Supplementary Fig. 27, alongside images relative to additional timepoints. Timestamps are reported with respect to the start of time-lapse imaging (Methods and Supplementary Table 6). **c**, Time-dependent mean of *μ*_CLD_, computed as discussed in Supplementary Methods 2. Data are shown as mean (solid line) ± s.d. (shaded region) from three fields of view within one sample. **d**, Scatter plot of condensate volume (*V*_C_) versus synthetic cell volume (*V*_SynCell_) for samples in **b**(i) and **b**(ii). Dashed lines indicate best fits to linear regression models, with 95% confidence intervals shown as shaded regions (Supplementary Methods 3). MLOs occupy 18.2 ± 0.5% and 15.3 ± 0.3% of the volume of the synthetic cells for A and B systems, respectively. **e**, Zoomed-in (top) and larger field-of-view (bottom) confocal micrographs depicting synthetic cells co-expressing A- and B-type condensates, with different template concentration ratios [A-T]/[B-T] (compare Fig. 2a). **f**, Distribution of the ratio between the radii of A and B MLOs (*r*_A_/*r*_B_) as a function of [A-T]/[B-T] for samples in **e** (Supplementary Methods 3). **g**, Percentage of synthetic cells containing a given number of A-type (top) or B-type (bottom) MLOs. The percentages of synthetic cells containing exactly one A and one B MLOs are 78.57%, 97.62%, 86.96%, 77.50% and 87.50% for [A-T]/[B-T] = 0.25, 0.50, 1, 2 and 4, respectively. Colour codes in **f** and **g** match those in **e**. Numbers in **f** and **g** indicate sampled synthetic cells. **h**, Confocal micrographs showing synthetic cells expressing three orthogonal MLO-forming RNA nanostars (A, B and C in Fig. 1) at different timepoints. Scale bars in **e**, bottom, and **h**, left and centre, are 150 μm. All other scale bars, 50 μm. Source data

Addressable RNA condensates could be extremely valuable to engineer compartmentalization in synthetic or living cells, where they could operate as MLOs capable of recruiting compounds and underpinning spatial separation of functionalities. To demonstrate this, we transcribed our condensates within synthetic cells constructed from water-in-oil (W/O) droplets (Fig. 3a). All designs formed a single spherical condensate in each synthetic cell (Fig. 3b and Supplementary Fig. 27), including design C that only generated extended networks in bulk. The different morphology is rationalized by noting that C aggregates need to relax over much smaller length scales within synthetic cells. Yet, shape relaxation was slower for C compared with A and B (Fig. 3c, and Supplementary Figs. 28 (top) and 29, and Supplementary Video 6 (top)). Polydispersity in condensate size reflects the variability in the size of synthetic cells, with the final volume of the condensates scaling linearly with that of the enclosing W/O droplet (Fig. 3d).

Control experiments with non-sticky designs revealed uniform fluorescence within the synthetic cells, confirming assembly specificity (Supplementary Figs. 28 (bottom) and 30, and Supplementary Video 6 (bottom)). Fluorimetry can be used to monitor RNA synthesis rates, as shown in Supplementary Fig. 31, where the delayed growth in the MGA signal (A component) is due to initial fluorophore partitioning in the oil phase^54,55^, rather than to a slower growth of the condensates (compare with Fig. 3c). Consistent with bulk data, initial transcription rates were found to scale (nearly) linearly with template concentration (Supplementary Figs. 32 and 33).

Supplementary Fig. 34 shows the time-dependent concentration of B nanostars during transcription transient (Supplementary Methods 5). The nanostar concentration exceeded 10 μM (or ~1 g l^−1^) within 1.5 h of the start of transcription (~15 min in Fig. 3b(ii); Supplementary Table 6), consistent with previous reports on DNA nanostars showing phase separation at concentrations as low as 0.25 μM (ref. ^40^) or ≤ 0.1 g l^−1^ (ref. ^56^).

We obtained synthetic cells with two distinct A and B MLOs, as shown in Fig. 3e, Supplementary Figs. 35–39, and Supplementary Videos 7 and 8 with microscopy, and Supplementary Fig. 40 with fluorimetry. In most cases, each synthetic cell contained one condensate of each type (Fig. 3g) and, like in bulk, we could control the relative size of the organelles by changing the template ratio (Fig. 3f). When including component C, we obtained three distinct phases (Fig. 3h, Supplementary Figs. 41–43 and Supplementary Video 9), exemplifying the possibility for scaling up the number of addressable organelles. Synthetic cells often failed to produce exactly three distinct MLOs, probably due to steric effects and the intrinsic slow relaxation of phase C and consistent with bulk experiments (Supplementary Figs. 25 and 26). When replacing component C with $\tilde{{{{\rm{C}}}}}$, which features identical KLs but lacks any FLAPs, synthetic cells with exactly three MLOs were more common (Supplementary Fig. 42), suggesting that aptamers affect the coarsening kinetics of the material.

**Fig. 4:**
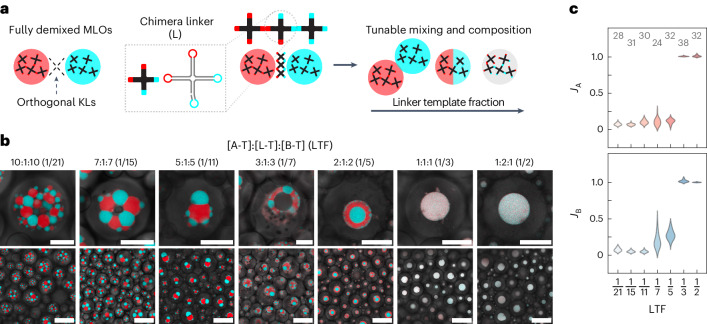
Controlling morphology and composition of MLOs with linker RNA nanostars. **a**, Chimeric RNA linker nanostars (L), with two A and two B KLs, enable control over mixing in systems of A and B nanostars by varying the relative concentration of the linker template L-T. **b**, Zoomed-in (top) and larger field-of-view (bottom) confocal micrographs, acquired after more than 48 h from the start of transcription, depicting synthetic cells producing A, B and L nanostars with different ratios between DNA templates ([A-T]:[L-T]:[B-T]). The LTF, shown in brackets, is computed as [L-T]/([A-T] + [L-T] + [B-T]). For LTF = 1/3 and 1/2, slight changes in condensate colour occur away from the confocal imaging plane, probably due to differences in the extinction coefficients of MG and DFHBI. Scale bars, 50 μm (top) and 150 μm (bottom). **c**, Distributions of mixing indices *J*_A_ and *J*_B_ of the MLOs (calculated as discussed in Supplementary Methods 3) and shown as a function of LTF for samples in **b**. Low *J*_A_ and *J*_B_ are indicative of purer A-rich and B-rich phases, while *J*_A_, *J*_B_ ≈ 1 indicate complete mixing of the two RNA species. Numbers indicate examined synthetic cells. Source data

We further expanded the possible organelle architectures in synthetic cells by introducing linker RNA nanostars, dubbed L, modulating the mixing between the A and B components. Similarly to the strategy demonstrated in ref. ^57^ with DNA constructs, nanostar L is ‘chimeric’, as it features two A-type and two B-type KLs (Fig. 4a). As shown in Fig. 4b, low fractions of the linker template L-T ([A-T]:[L-T]:[B-T] = 10:1:10, or linker template fraction (LTF) = 1/21) produced grape-like clusters, blocking the relaxation of A and B domains into two large condensates (Supplementary Figs. 44–46). Arrested coarsening is arguably due to interphase adhesion limiting the ability of the condensates to slide past each other, as noted for linker-free A–B systems with crowding agents (Supplementary Fig. 12). At higher LTFs, bigger A- and B-rich domains formed, with Janus-like morphologies emerging at [A-T]:[L-T]:[B-T] = 5:1:5 (LTF = 1/11) (Supplementary Fig. 46, and Supplementary Videos 10 and 11). Here we note the occasional formation of a cavity in the interphase contact region, hinting at an uneven linker distribution. For [A-T]:[L-T]:[B-T] = 3:1:3 (LTF = 1/7) we observe hollow, capsule-like organelles for most of the larger synthetic cells (Supplementary Videos 11 and 12, and Supplementary Fig. 46). Composition [A-T]:[L-T]:[B-T] = 2:1:2 (LTF = 1/5) produced Russian-doll morphologies with an A-rich outer shell, while single-phase condensates were observed for LTFs ≥ 1/3.

The trends observed in condensate morphology are broadly consistent with a decrease in the interfacial tension between A- and B-rich phases (*γ*_AB_) with increasing LTF, as observed for equilibrium assembly of DNA nanostars^57^. Adhering de-mixed droplets (LTF = 1/21 to 1/11), are indeed expected at equilibrium when *γ*_AB_ ≈ *γ*_A_ ≈ *γ*_B_, where *γ*_A_ (*γ*_B_) is the interfacial tension between the A-rich (B-rich) phase and the surrounding buffer^2^. The Russian-doll morphology (LTF = 1/5) should emerge for *γ*_A_ < *γ*_AB_ < *γ*_B_, with the evidence that *γ*_A_ < *γ*_B_ being consistent with trends seen in melting temperatures (Fig. 1c), while full mixing (LTF ≥ 1/3) should occur when *γ*_AB_ ≈ 0 (ref. ^57^). However, some morphological features, including the cavities seen for LTF = 1/11 and 1/7 and the outer layer of small B-rich domains seen for LTF = 1/5, are not expected at equilibrium, hinting that these may constitute metastable states emerging from isothermal co-transcriptional assembly.

Confocal projections reveal a curved morphology for the clusters formed at LTF = 1/21, ascribed to sedimentation within the W/O droplet (Supplementary Fig. 46). In all other conditions, the compact condensates appeared unaffected by substrate curvature.

The abundance of linkers also influences the degree of mixing between the two phases, which we quantified with indices *J*_A_ and *J*_B_. Index *J*_A_ (*J*_B_) was computed as the ratio between the fluorescence intensity from nanostars A (B) in the B-rich (A-rich) phase over the signal in the A-rich (B-rich) phase (Supplementary Methods 3). We observed limited mixing (*J*_A_, *J*_B_ ≪ 1) for low LTF, followed by a moderate increase and by an abrupt jump to *J*_A_, *J*_B_ ≈ 1 upon reaching the threshold for complete mixing (Fig. 4c). A similarly sharp mixing transition was noted for DNA nanostars, remarkably occurring at similar fractions of tetravalent linkers^58^.

## Selective protein capture in RNA condensates

**Fig. 5:**
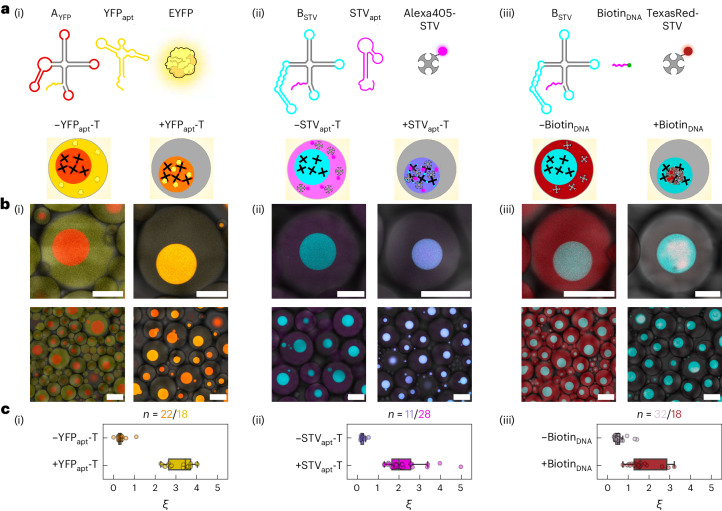
Selective protein capture in designer RNA MLOs. **a**, RNA-nanostar designs are modified to include a single-stranded 5′ overhang that can connect to a protein-binding moiety. Design A_YFP_ is identical to nanostar A (Fig. 1) but can connect to YFP-binding aptamer (YFP_apt_) (i). Created with BioRender.com. Design B_STV_ is identical to B but can connect to either an STV-binding aptamer (STV_apt_) (ii) or a biotinylated DNA oligonucleotide (Biotin_DNA_) (iii). The templates of protein-binding aptamers (YFP_apt_-T and STV_apt_-T) are transcribed in synthetic cells alongside the associated RNA-nanostar templates (A_YFP_-T and B_STV_-T, respectively), while pre-synthesized Biotin_DNA_ is encapsulated alongside template B_STV_-T. **b**, Diagrams (top) and confocal micrographs (bottom) of synthetic cells expressing RNA MLOs in the absence (left) or in the presence (right) of protein-binding moieties for the systems presented in **a**. When protein-binding aptamers are expressed or the biotinylated DNA oligonucleotide is included, target proteins are recruited in the MLOs. Scale bars, 50 μm (top) and 150 μm (bottom). **c**, Protein partitioning parameter, *ξ*, calculated from confocal micrographs in **b** as the ratio between the fluorescence signal of the target proteins recorded within and outside the MLOs for individual synthetic cells (Supplementary Methods 3). Data are shown for the three systems in **a** and **b**, with and without protein-binding moieties. In the box plots, the central line marks the median, the box marks the Q1 (first quartile) – Q3 (third quartile) interquartile range (IQR) and the whiskers enclose data points within Q1 − 1.5 × IQR and Q3 + 1.5 × IQR. Data points are relative to individual synthetic cells from a single field of view of one imaged sample ((i), (ii) and (iii), left) or two fields of view from two technical replicates ((iii), right). All data points are shown except for four outliers with *ξ* > 5 in (i) and (iii), omitted for ease of visualization. Source data

While Figs. 1–4 demonstrate that RNA condensates can selectively sequester small molecules—the fluorophores associated with MGA and BrA—imitating natural MLOs requires capturing larger and functional macromolecules, particularly proteins. To this end, we modified designs A and B to include a 5′ overhang, to which a protein-binding RNA aptamer can connect via base pairing (Fig. 5a(i),(ii)). Nanostructures A_YFP_ and B_STV_ were thus designed to connect to a yellow fluorescent protein (YFP)-binding aptamer (YFP_apt_)^59^ and a streptavidin (STV)-binding aptamer (STV_apt_)^60^, respectively (Supplementary Fig. 47).

For both designs, co-expressing the modified nanostars and their partner aptamers from distinct templates in synthetic cells (Supplementary Fig. 48) led to the target proteins (enhanced yellow fluorescent protein (EYFP) and Alexa Fluor 405 streptavidin (Alexa405-STV) conjugate) readily partitioning within the formed MLOs (Fig. 5b(i),(ii), Supplementary Figs. 49 and 50, and Supplementary Video 13). Omitting the aptamer led to the target proteins remaining in the lumen of the synthetic cell. Protein partitioning was quantified through the parameter *ξ*, calculated as the ratio between the fluorescence intensity of the protein recorded within or outside the MLOs (Supplementary Methods 3). When protein-binding aptamers were present, the median *ξ* was ~3.5 and ~2 for YFP and STV, respectively, while it fell below 0.5 when no aptamers were present (Fig. 5c(i),(ii)). The significant anti-partitioning noted in the absence of aptamers is probably a consequence of excluded volume interactions between RNA and proteins. Indeed, the condensate mesh size, estimated as twice the RNA-nanostar arm length (~15.7 nm; Supplementary Methods 5), is comparable with the hydrodynamic diameters of the STV (6.4 nm) and EYFP (5 nm)^61^. Both STV and EYFP have mildly acidic to neutral isoelectric points^62,63^, hence Coulomb repulsion towards RNA may also enhance anti-partitioning.

As an alternative strategy for STV capture, we replaced STV_apt_ with a biotinylated DNA oligonucleotide complementary to the overhang in B_STV_ (Fig. 5a(iii), Supplementary Figs. 49 and 50 and Supplementary Video 13). With this approach, TexasRed streptavidin (TexasRed-STV) conjugate was distributed non-uniformly within the condensates, forming irregularly shaped clusters with solid-like appearance (Fig. 5b(iii) and Fig. 5c(iii)), reminiscent of multi-phase cellular condensates^64–66^. The non-uniform protein distribution probably results from the finite amount of the biotinylated DNA anchor available, which is all sequestered at early transcription stages and thus accumulates at the centre of the condensates. The solid-like look of the protein-rich material may be a consequence of the tetravalent STV strongly cross-linking multiple RNA nanostars, making the material more viscous.

## Conclusion

Our platform enables the expression of synthetic condensates and MLOs with prescribed size, number, morphology and composition, and able to capture small guest molecules and proteins. The elementary building blocks, RNA nanostars, were designed utilizing the rule-based approaches of nucleic acid nanotechnology, which provide extensive opportunities for updates aimed at programming arbitrary characteristics of the designer MLOs. Among the many features that can be straightforwardly controlled are nanostar valency, flexibility and arm length, all known to predictably influence self-assembly in analogous DNA systems^21,23,28,40,67^. Control over condensate size could be achieved by co-transcribing surface-passivating RNA constructs^68,69^. Further aptamers might be embedded to recruit molecular guests, including enzymes and metabolites, while ribozymes^70^ could confer catalytic properties to the MLOs. Because the condensates are transcribed from DNA templates, their formation could be controlled through standard transcription regulation pathways in both synthetic cells and living cells. Owing to their open-ended programmability, we expect that the RNA-nanostar condensates will constitute a valuable solution for the toolkit of synthetic biology.

## Methods

### Sequence design

Four-armed RNA nanostars with 25 base pair (bp)-long arms separated by one unpaired uracil residue were designed starting from the sequences of previously reported RNA junctions^71^ and fluorogenic MG^25,26^ and Broccoli^27^ RNA aptamers. Three main nanostar variants, namely A, B and C, were designed to bind identical motifs, while being non-interacting towards each other. Intra-population interactions were guaranteed by palindromic KLs. KL A (5′-AUCGCGAAA-3′) was adapted from the KL domain in the bottom right arm of the T4 tetrahedron in ref. ^71^, by making it palindromic. Asymmetrical flanking bases (5′-A…AA-3′) were introduced due to their presence, albeit in reverse order, in the KL domains found in the Lai variant of the human immunodeficiency virus-1 dimerization initiation sequence (HIV-1 DIS)^72,73^. KLs B and C were designed to match the interaction strength of KL A. This was achieved by computationally generating, using Python3, all possible palindromic, 6 nt sequences with the same GC content as KL A. The resulting set was filtered to exclude all sequences with more than two overlapping nucleotides with KL A. Among these, KL C was selected as 5′-AGGUACCAA-3′. To determine KL B, the constraint was relaxed to allow a maximum of 3 overlapping nucleotides with both KL A and KL C. Among these, KL B was chosen as 5′-AGUCGACAA-3′—a sequence similar to the Mal variant of the HIV-1 DIS KL (5′-GUGCAC-3′)^72^. Design $\tilde{{{{\rm{C}}}}}$ is similar to C and shows the same KL domains, but lacks FLAPs. The minimum free energy configuration of all designs was evaluated using NUPACK (default Serra and Turner, 1995 parameters and 1 M Na^+^)^74^^,75^. Kinefold^76^ was used to test co-transcriptional folding. All designs were tested via batch jobs, and further considered only if the helix tracing graph showed correct folding order and reasonable stability of each helix. All nanostar sequences are provided in Supplementary Table 1. Sequences for the coding/non-template DNA strands were obtained by adding a prefix comprising the T7 promoter (5′-TTCTAATACGACTCACTATA-3′, 17 nt consensus T7 promoter underlined) to the equivalent DNA sequence of the RNA nanostructures^71^ (Supplementary Table 2). DNA primers for PCR amplification of the templates were designed aiming for 40−60% GC content and length between 18 nt and 26 nt (Supplementary Table 3). DNA primers were verified using NUPACK^74^ and the NEB melting temperature calculator (https://tmcalculator.neb.com/#!/main) for use with Q5 High-Fidelity DNA Polymerase under standard 500 nM primer concentration.

### Materials

DNA primers were purchased from and purified by Integrated DNA Technologies via standard desalting. Unless otherwise specified, dsDNA templates were purchased from Integrated DNA Technologies as gBlocks. Only the shorter STV_apt_-T was purchased as a double-stranded Ultramer. All DNA strands were received lyophilized and reconstituted at 100 μM. DNA primers were reconstituted in nuclease-free water (UltraPure DNase/RNase-free distilled water, Invitrogen), while gBlock DNA templates were reconstituted in syringe-filtered TE buffer (10 mM Tris, 1 mM EDTA, pH 8.0), obtained by diluting Tris-EDTA 100× (Sigma-Aldrich) in nuclease-free water. DNA primer concentration was determined by measuring absorbance at 260 nm (average of 5 repeated measurements) using a Thermo Scientific Nanodrop One Microvolume ultraviolet–visible spectrophotometer using extinction coefficients provided by the supplier. Primers were then diluted to 10 μM in nuclease-free water as per PCR kit instructions. MG chloride and DFHBI were purchased from Sigma-Aldrich. The as-received powders were dissolved in nuclease-free water and DMSO to produce 1 mM and 10 mM stock solutions, respectively. The 10 mM DFHBI solution was then diluted to 1 mM in nuclease-free water. The resulting 1 mM MG and DFHBI solutions were stored at 4 °C and −20 °C, respectively. Recombinant EYFP was purchased from RayBiotech, resuspended at 0.67 mg ml^−1^ in nuclease-free water and stored at −80 °C. EYFP was used within 2 days from reconstitution to prevent aggregation. TexasRed-STV conjugate was purchased from Sigma-Aldrich (CalBioChem), already resuspended at 1 mg ml^−1^ in 50 mM bicarbonate-borate buffer, 0.9% NaCl, 5 mg ml^−1^ BSA, pH 8.1, and stored at 4°C. Alexa405-STV conjugate was purchased from Invitrogen, resuspended at 1 mg ml^−1^ in PBS, pH 7.2 (Gibco) with addition of 5 mM sodium azide (0.1 M solution, Sigma-Aldrich), and stored at −20 °C. The dsDNA ladder for electrophoresis (GeneRuler Ultra Low Range DNA Ladder) was purchased from Thermo Scientific. The ssRNA ladder (RiboRuler Low Range ssRNA Ladder) and 2× RNA Gel Loading Dye were purchased from ThermoFisher.

### PCR amplification and purification of DNA templates

Amplification of gBlock DNA templates was carried out using Q5 High-Fidelity DNA Polymerase (New England Biolabs). PCR mixtures were prepared on ice, with 10 ng DNA template per mixture, and annealed in a Bio-Rad C1000 Touch Thermal Cycler according to the following protocol: pre-heating at 98 °C; initial denaturation at 98 °C for 30 s; 30 amplification cycles (98 °C for 10 s, 64–65 °C for 20 s depending on primer melting temperature, 72 °C for 7 s); final extension at 72 °C for 2 min; hold at 4 °C. Samples were stored at 4 °C and gel-purified within 1 week. Purification was carried out using a 2% w/v agarose (Sigma-Aldrich) gel, prepared in Tris-borate-EDTA (TBE) 1× buffer (from TBE 10×, Thermo Scientific) with GelRed nucleic acid gel stain (3×, Biotium) and run at 120 V for 90 min (Supplementary Fig. 2). Gels were imaged using a Syngene G:BOX Chemi XRQ gel documentation system. One PCR reaction (50 μl + 10 μl TriTrack DNA Loading Dye 6×, Thermo Scientific) was loaded in each well, and bands were cut using a scalpel under the ultraviolet illumination. Gel bands were loaded in pairs in 2 ml Eppendorf tubes, treated by adding 4 μl of Monarch Gel Dissolving Buffer (New England Biolabs) per mg of gel, and incubated at 50 °C until complete dissolution. The obtained mixtures were purified using the Monarch PCR & DNA Cleanup Kit (New England Biolabs). Elution was performed with 12–14 μl per gel-band pair. The concentration of purified DNA templates was determined by measuring absorbance at 260 nm (average of 3 repeated measurements) using a NanoDrop One.

### RNA transcription in bulk

Transcription was carried out using the CellScript T7-FlashScribe Transcription Kit. The final reaction mixture contained T7 RNAP in the proprietary transcription buffer, complemented by 9 mM of each ribonucleotide triphosphate, 0.05 units per μl of RNase inhibitor and 10 mM dithiothreitol. Unless otherwise specified, DNA templates were added to an overall concentration of 40 nM and both MG and DFHBI dyes were added to all transcription mixtures (even samples lacking the corresponding aptamer) in proportions equal to 1 μl of 1 mM dye:20 μl mixture, yielding a final concentration of approximately 45.45 μM for each dye. Unless otherwise specified, samples were loaded in rectangular glass capillaries (either 0.20 mm × 4.00 mm × 50 mm or 0.40 mm × 4.00 mm × 50 mm, VitroCom) sealed and glued on a glass coverslips (24 mm × 60 mm, Menzel Gläser) via a 2-component epoxy (Araldite Rapid). To avoid the glue coming in contact with the sample, the sides of the capillary were capped with mineral oil. Glue was allowed to set for 30 min, during which samples were kept in a dark environment at room temperature.

### RNA transcription in synthetic cells and protein capture

Synthetic cells were generated by encapsulating the in vitro transcription mixture described above within W/O droplets^77^. Briefly, 22–23 μl of transcription mixture were added on top of 90 μl of 2% w/w Pico-Surf (Sphere Fluidics), a biocompatible surfactant, in Novec 7500, a fluorinated oil, within an Eppendorf tube. The resulting mixture was vortexed at 2,500 rpm for 30 s and then left to equilibrate for 1–2 min before extracting the top layer containing the synthetic cells. For protein capture experiments, unless otherwise specified, the volume of transcription mixtures was increased to 23 μl to accommodate EYFP, TexasRed-STV or Alexa405-STV, each at a final concentration of 1.25 μM. For control experiments in the absence of target proteins the transcription mixture volume was kept to 22 μl. In assays relying on protein-binding aptamers (YFP_apt_, STV_apt_), the total DNA template concentration was kept equal to 40 nM, and the composition ratio was chosen to be [nanostar DNA template]/[aptamer DNA template] = 3. For TexasRed-STV capture assays via Biotin_DNA_, [B_STV_-T] was similarly kept at 30 nM, while [Biotin_DNA_] was chosen to be 10 μM, yielding an approximately 8× excess of biotin compared with streptavidin. MG was omitted in assays including TexasRed-STV due to fluorescence emission overlap. Unless otherwise specified, samples were loaded in capillaries as for bulk samples, but omitting mineral oil capping.

### Effect of buffer exchange on condensate stability

For buffer-exchange experiments (Supplementary Fig. 11), bulk transcription samples (20 μl per sample) were prepared as described above and loaded in 384-well plates (black, Greiner Bio-One) to enable buffer exchange. The DNA template concentration was reduced to 2 nM to account for the reduced bottom-surface-to-volume ratio of the microplate wells compared with the capillary chambers, avoiding the formation of extremely large condensates upon sedimentation. Microplates were sealed with an adhesive aluminium film. Samples were incubated using a custom-made microplate heated stage with temperature set at 30 °C for the plate chamber and at 35 °C for the lid, and then imaged after 24 h. Samples underwent buffer exchange with either PBS 1× pH 7.4 (diluted from 10× PBS, Invitrogen), TE 1× (diluted from 100× TE, ThermoFisher) supplied with 5 mM MgCl_2_ (Sigma-Aldrich) pH 8.0 or TE 1× supplied with 10 mM MgCl_2_ pH 8.0 via four consecutive washes, separated by 30 min intervals, during which the samples were kept at 30 °C. For the first wash, 70 μl of the desired buffer were added to the sample well. For remaining washes, 70 μl supernatant were removed before adding an equal volume of fresh buffer. Samples were imaged after the final wash and after an additional 24 h incubation at 30 °C.

### Effect of crowding agents

To test the effect of crowding agents (Supplementary Fig. 12), bulk samples were prepared as described above. To reach the indicated final volume, samples were supplemented with either RNase-free water (for control samples, ‘no PEG’) or PEG 200 (Sigma-Aldrich) at a final concentration of 25% v/v. Samples were incubated in a Bio-Rad C1000 Touch Thermal Cycler at 30 °C (heated lid at 35 °C) for 24 h before imaging.

### FRAP

For FRAP experiments conducted on FLAPs (Supplementary Fig. 18(i)), bulk samples were prepared as described above. For FRAP conducted using the covalently linked fluorescein-12-UTPs (Supplementary Fig. 18(ii)), bulk samples were prepared as described above with the difference that uridine triphosphate (UTP) concentration was reduced from from 9 mM to 8.95 mM and 50 μM of fluorescein-12-UTP (Sigma-Aldrich) were included for labelling, corresponding to ~0.55% of the total UTP content. MG and DFBHI were not included. Samples were incubated in a Bio-Rad C1000 Touch Thermal Cycler at 30 °C (heated lid at 35 °C) for 24 h before imaging.

### Fluorimetry

For fluorimetry experiments in bulk and in synthetic cells, including excitation/emission scans reported (Supplementary Fig. 1) and kinetics assays (Supplementary Figs. 13–15, 31–33, 40 and 43), samples were prepared as discussed above and loaded in transparent UV-Star 384-well plates (Greiner Bio-One).

### Epifluorescence imaging

Time-lapse epifluorescence imaging of RNA transcription, in bulk and in synthetic cells, was performed on a Nikon Eclipse Ti2-E inverted microscope with Perfect Focus System (PFS), equipped with Plan Apochromat *λ* 10× (numerical aperture (NA) 0.45, working distance (WD) 4,000 μm) and *λ* 20× (NA 0.75, WD 1,000 μm) objective lenses, a Hamamatsu Orca-Flash 4.0 v3 camera, and a Lumencor SPECTRA X light-emitting diode engine. The following SPECTRA X light-emitting diodes were used to excite the corresponding fluorophores or FLAPs: 395 nm for Alexa405-STV, 470 nm for DFHBI/BrA, 550 nm for EYFP, 575 nm for TexasRed-STV, and 640 nm for MG/MGA. Samples, enclosed in glass capillaries and glued to a microscope coverslip (see above), were taped to a Peltier-controlled copper temperature stage (Temikra). Imaging was automated via the ND acquisition module of Nikon’s NIS software, with PFS enabled to ensure constant *z*-height during the time-lapse. Three non-overlapping fields of view per capillary (sample) were imaged. Epifluorescence *z*-stacks (120–160 μm *z*-height, distributed from −20/−60 μm to +100 μm around the PFS plane, with a 3.5-4 μm step depending on the run) were captured at each timepoint.

For condensate formation time-lapses, both in bulk and in synthetic cells, the temperature was set to 30 °C. Samples were imaged every 15 min for 10 h, and every 30 min for further 38 h. For binary and ternary systems in bulk, automated acquisition terminated at 42 h and data at 48 h were collected manually.

For melting-temperature determination experiments (Supplementary Fig. 8), temperature was set to 25 °C and increased by 1 °C every 15 min up to 75 °C. Samples were imaged after 10 min of hold at each temperature.

Due to the time required for sample preparation and set-up, imaging started 1–2 h after mixing the DNA templates with the rest of the transcription mixtures (Supplementary Tables 5 and 6). Micrographs and videos have been labelled according to imaging time, with time 0 referring to the start of the imaging run, rather than to the start of transcription. When comparing samples from different runs in the same figures, timepoints have been aligned to reflect any delays in the run starting times (as in Fig. 3b, and Supplementary Figs. 44, 45, 49 and 50). Conversely, videos have all been labelled independently, that is, relative to the start of their specific run.

### Confocal imaging

As well as FRAP experiments, laser scanning confocal imaging was performed on a Leica Stellaris 8 (DMi8 CS Premium) inverted microscope. The microscope was equipped with a solid-state 405 nm laser as well as a white-light laser (440–790 nm). The following objective lenses were used: HC PL APO CS2 10× DRY (NA 0.40) and HC PL APO CS2 20× (NA 0.75) DRY. For Alexa405-STV, the 405 nm laser was used and emission was recorded around 421 nm. The white-light laser was used for all other dyes with the following excitations/emission wavelengths: DFHBI/BrA, 447/501 nm; EYFP, 514/527 nm; TexasRed-STV, 595/615 nm; MG/MGA, 628/650–660 nm. A line-sequential illumination mode was adopted; for example, bright-field, 405 nm and 628 nm in sequence 1, 447 nm in sequence 2, 514 nm in sequence 3. The pinhole was set to 1 airy unit. Line-averaging was enabled and set to 2–3. The scan mode along the *x* direction was selected to be bidirectional after phase calibration. The scanning speed was set to 400 Hz for high-resolution stills (4,096 px × 4,096 px or 8,192 px × 8,192 px) captured with the 10× or 20× lens, and 1,000 Hz for zoomed-in *z*-stacks acquired with the 20× lens. For the latter, the zoom factor was tuned to select a single condensate or droplet, and top/bottom planes were manually tuned, with a Nyquist optimized *z*-step. Unless otherwise stated, all reported confocal micrographs are pristine. Two-dimensional orthogonal cross-sections (*XY*, *XZ*, *YZ*) and volume three-dimensional renderings (Supplementary Fig. 46) were generated from *z*-stacks using ‘Sections’ in the three-dimensional module of Leica Application Suite (LAS) X. Clipping of three-dimensional renderings in Supplementary Video 11 were produced via the ‘Movie Editor’ in LAS X, converted to AVI using ffmpeg, collated in FIJI and finally re-exported in MP4 using Permute3.

### Gel electrophoresis on RNA transcripts

Samples were prepared following the bulk transcription protocol described above, but reducing the sample volume to 10 μl. No MG or DFHBI dyes were added. The samples were incubated in a Bio-Rad C1000 Touch Thermal Cycler at 30 °C (heated lid at 35 °C) for 16–18 h, and then treated with 0.5 μl of the DNase I solution provided with the transcription kit (1 unit per μl) for 30 min at 37 °C (heated lid at 40 °C).

For non-denaturing agarose gel electrophoresis (AGE) (Supplementary Fig. 4), after incubation, samples were diluted 3× with RNase-free water and mixed with 3 μl of 60% glycerol (used instead of loading dyes). Six microlitres of each diluted sample was loaded into the gel wells. Ten microlitres RiboRuler Low Range ssRNA Ladder (ThermoFisher) was loaded in the leftmost lane. For the control DNA nanostars (Supplementary Table 4), 10 μl of 0.7 μM DNA sample (annealed in the Bio-Rad C1000 Touch Thermal Cycler) were loaded in the rightmost lane. Agarose gels were prepared at 2% w/v agarose (Sigma-Aldrich) in TBE 1× buffer (from TBE 10×, Thermo Scientific), with the addition of GelRed nucleic acid gel stain (3×, Biotium). Gels were run at 120 V for 45 min.

For denaturing polyacrylamide gel electrophoresis (PAGE) (Supplementary Fig. 3), RNA transcripts were prepared and treated with DNase I as for native AGE, then diluted 50× with RNase-free water. Sample (4 μl) and RiboRuler Low Range ssRNA Ladder (2.5 μl) aliquots were each mixed with equal volumes of 2× RNA Gel Loading Dye (ThermoFisher), before incubation in a Bio-Rad C1000 Touch Thermal Cycler at 70 °C (heated lid at 80 °C) for 10 min. All samples were promptly transferred on ice before loading onto the gel. Eight microlitres of each sample and 5 μl of RiboRuler Low Range ssRNA Ladder were loaded into the wells of a 7 M urea, 8% polyacrylamide gel. The gels were prepared by combining 3.7 ml 30% w/v acrylamide/bis-acrylamide partitioned solution (29:1, Sigma-Aldrich), 4.0 ml 10× TBE buffer (ThermoFisher), and 6.3 g urea (Sigma-Aldrich), to achieve a mixture with 7 M final urea concentration. To obtain a 1.5 mm 8% polyacrylamide gel, this volume was adjusted to 14 ml using UltraPure RNase-free water in a 50 ml Falcon tube. Following the addition of 75 μl of 10% w/v ammonium persulfate (Sigma-Aldrich) and 15 μl *N*,*N*,*N*′,*N*′-tetramethylethylenediamine (Sigma-Aldrich), the tube was inverted several times. The final mixture was allowed to polymerize for 20 min in an assembled gel electrophoresis cassette (Bio-Rad). Gels were run at 150 V for 60 min, followed by post-staining with 1× SYBR Gold Nucleic Acid Gel Stain (diluted from 10,000× concentrate in DMSO, ThermoFisher) in 1× TBE buffer for 15 min.

Gels were imaged using a Syngene G:BOX Chemi XRQ gel documentation system.

### Statistics and reproducibility

No statistical method was used to determine sample size. The experiments were not randomized. The investigators were not blinded to allocation during experiments and outcome assessment. No data were excluded except (in limited cases) when removing artefacts of incorrect segmentation of microscopy images, as specified in Supplementary Methods 2 and 3. Several control experiments were executed (Supplementary Information) and found to be consistent. Information on repeats is provided in the relevant figure captions. No reproducibility issues emerged.

### Reporting summary

Further information on research design is available in the Nature Portfolio Reporting Summary linked to this article.

## Online content

Any methods, additional references, Nature Portfolio reporting summaries, source data, extended data, supplementary information, acknowledgements, peer review information; details of author contributions and competing interests; and statements of data and code availability are available at 10.1038/s41565-024-01726-x.

## Supplementary information

Supplementary Information Supplementary Methods 1–5, Tables 1–6, Figs. 1–50 and uncropped/unprocessed images of gel electrophoresis.

Reporting Summary

Supplementary Video 1 Self-assembly of single RNA species systems in bulk.

Supplementary Video 2 Melting of condensates from single RNA species in bulk.

Supplementary Video 3 Self-assembly of binary RNA systems with varying DNA template ratio in bulk.

Supplementary Video 4 Self-assembly of binary RNA systems with non-sticky species in bulk.

Supplementary Video 5 Self-assembly of ternary RNA system in bulk.

Supplementary Video 6 Self-assembly of single RNA species systems in synthetic cells.

Supplementary Video 7 Self-assembly of binary RNA systems with varying DNA template ratio in synthetic cells.

Supplementary Video 8 Self-assembly of binary RNA systems with non-sticky species in synthetic cells.

Supplementary Video 9 Self-assembly of ternary RNA systems in synthetic cells.

Supplementary Video 10 Self-assembly of RNA mixtures with programmable linker-induced mixing in synthetic cells.

Supplementary Video 11 Three-dimensional rendering and clipping showcasing the internal structure of condensates formed in A:L:B systems with LTF = 1/11, 1/7 and 1/5.

Supplementary Video 12 Confocal *z*-stack showcasing the internal structure of condensates formed in A:L:B systems with LTF = 1/7.

Supplementary Video 13 Self-assembly of protein-capturing RNA organelles.

## Source data

Source Data Fig. 1 Statistical source data for Fig. 1c,d(i),(ii),e(i),(ii),f. **c**, Melting temperatures of bulk condensates (A, B, C). **d**, (i) Mean of CLD, mean ± s.d. of mean replicates of CLD versus time. (ii) Mean condensate number per field of view, mean condensate number per field of view ± s.e. of the mean versus time. **e**, Aspect ratios and exponentially fitted aspect ratios from coalescence events versus time. **f**, Coalescence time constants (*τ*) and fitted *τ* versus initial sizes.

Source Data Fig. 2 Statistical source data for Fig. 2c,d. **c**, Mean of CLD, mean ± s.d. of mean replicates of CLD versus time. **d**, Radii of A/B condensates for different [A-T]/[B-T] template ratios.

Source Data Fig. 3 Statistical source data for Fig. 3c,d,f,g. **c**, Mean of CLD, mean ± s.d. of mean replicates of CLD versus time, **d**, Organelle volumes versus enclosing droplet/synthetic cell volumes. **f**, Condensate/organelle radial ratio (*r*_A_/*r*_B_) for different [A-T]/[B-T] template ratios. **g**, Fraction of synthetic cells displaying 1, 2, 3 A or B organelles (Figure_3g.csv) and raw counts of A/B organelles in examined droplets per template ratio (Figure_3g_rawcounts.csv).

Source Data Fig. 4 Statistical source data for Fig. 4c. Linker-induced mixing indices (A in B, B in A, average) for different LTFs.

Source Data Fig. 5 Statistical source data for Fig. 5c. Protein fluorescence intensity ratio within organelle and outside (in the droplet lumen) for various protein-capturing systems in the presence/absence of protein-capturing construct (or template coding for it).

## Data Availability

The raw data underpinning this publication are available, free of charge, at 10.17863/CAM.108563. For large microscopy datasets (time-lapses, *z*-stacks), a representative selection of all the data is provided after binning and time downsampling due to space limitations on the repository. The full dataset is available from the corresponding author. Oligonucleotide sequences generated for this work are provided in the Supplementary Tables 1–4. Source data are provided with this paper.

## Code availability

The data analysis code used in this publication is available at https://github.com/ld389/Fabrini-2024-Nat-Nanotechnol.
